# Mouse Models of Anemia of Cancer

**DOI:** 10.1371/journal.pone.0093283

**Published:** 2014-03-28

**Authors:** Airie Kim, Seth Rivera, Dana Shprung, Donald Limbrick, Victoria Gabayan, Elizabeta Nemeth, Tomas Ganz

**Affiliations:** 1 Department of Medicine, David Geffen School of Medicine, University of California Los Angeles, Los Angeles, California, United States of America; 2 Department of Pathology, David Geffen School of Medicine, University of California Los Angeles, Los Angeles, California, United States of America; Lady Davis Institute for Medical Research/McGill University, Canada

## Abstract

Anemia of cancer (AC) may contribute to cancer-related fatigue and impair quality of life. Improved understanding of the pathogenesis of AC could facilitate better treatment, but animal models to study AC are lacking. We characterized four syngeneic C57BL/6 mouse cancers that cause AC. Mice with two different rapidly-growing metastatic lung cancers developed the characteristic findings of anemia of inflammation (AI), with dramatically different degrees of anemia. Mice with rapidly-growing metastatic melanoma also developed a severe anemia by 14 days, with hematologic and inflammatory parameters similar to AI. Mice with a slow-growing peritoneal ovarian cancer developed an iron-deficiency anemia, likely secondary to chronically impaired nutrition and bleeding into the peritoneal cavity. Of the four models, hepcidin mRNA levels were increased only in the milder lung cancer model. Unlike in our model of systemic inflammation induced by heat-killed *Brucella abortus*, ablation of hepcidin in the ovarian cancer and the milder lung cancer mouse models did not affect the severity of anemia. Hepcidin-independent mechanisms play an important role in these murine models of AC.

## Introduction

Anemia is a remarkably common complication of cancer, occurring in >30% of untreated patients [1]. The incidence of anemia is further increased by cancer treatment, including chemotherapy and radiation. Anemia of cancer (AC) correlates with poor WHO performance status and quality of life [2], [3]. Effective treatment of AC decreases subjective fatigue in cancer patients [4]. In addition to the effects on quality of life, the presence of AC may be an independent negative prognostic factor [5].

The etiology of AC is multifactorial and likely varies with the origin of the primary tumor, as well as the chronicity of the disease process. Important contributing factors include iron deficiency secondary to bleeding or nutritional deficiencies [6], hemolysis due to immune phenomena, activation of clotting cascades or abnormal tumor-associated vasculature [7], impaired erythropoiesis secondary to inadequate erythropoietin production or depressed response of the erythroid marrow to erythropoietin [8], and direct invasion of the marrow by tumor cells with disruption of the erythropoietic environment. A particularly important contributor to AC is inflammation, as AC shares many of the characteristics of anemia of inflammation (AI). AI is a normocytic, normochromic anemia with a shortened erythrocyte lifespan and depressed erythropoiesis. AI is also characterized by a derangement of systemic iron homeostasis characterized by hypoferremia with intact iron stores [9] and decreased availability of iron for erythrocyte production.

Hepcidin, a 25-amino acid peptide hormone produced primarily by hepatocytes [10], is the primary regulator of iron homeostasis in health and during inflammation [11]. Hepcidin acts by binding to ferroportin, the sole known cellular iron exporter, displayed on the surface of cells that supply iron to plasma: macrophages, hepatocytes and enterocytes. Hepcidin binding to ferroportin leads to ferroportin endocytosis and degradation [12], limiting the flow of iron into plasma and thus reducing iron availability for erythropoiesis. During inflammation, hepcidin expression is strongly induced, largely by IL-6 [13] via the JAK-STAT pathway [14]–[16] and this hepcidin excess is thought to contribute to the development of AI. Interestingly, IL-6 has been noted to be increased in multiple tumor types, including lung cancer [17] and ovarian cancer [18]. The extent to which increased hepcidin production contributes to AC has not been directly tested by genetic ablation of hepcidin, partially because of the paucity of mouse models of AC.

Despite the high prevalence of AC with its negative effects on quality of life and survival, few animal models have been developed for its study. We have characterized four syngeneic C567BL/6 mouse models of cancer: two rapidly-growing models of lung cancer, one rapidly-growing model of melanoma, and one slow-growing model of ovarian cancer. After a comprehensive examination of hematologic, iron, and inflammatory parameters of these models, we investigated the extent of hepcidin involvement in the development of AC in two of these models by comparing C57BL/6 mice to hepcidin knockout mice on the same strain background.

## Materials and Methods

### Ethics Statement

All animal work and care were performed under the guidelines of the University of California, Los Angeles (UCLA) Chancellor's Animal Research Committee (ARC). Specific approval for the mouse experiments was obtained with the protocol #2008-129-12 titled “The role of IL-6 and hepcidin in anemia of cancer.” All reasonable efforts were made to ameliorate suffering, including anesthesia for painful procedures.

### Animal models of anemia of cancer

All animal studies were approved by the Animal Research Committee at University of California, Los Angeles (UCLA). 6-week-old C57BL/6J mice were obtained from Charles River Laboratories (Wilmington, MA) or The Jackson Laboratories (Bar Harbor, ME). For the three rapidly-growing cancer models, male and female wild-type (WT) mice were placed on an iron-sufficient diet (20 ppm iron, Harlan Teklad, Indianapolis, IN) for 2 weeks before injection of tumor cells or saline. The same diet was used through the remainder of the experiment. This dietary conditioning was applied because the high iron content of standard chow (approximately 7–8 times the daily requirement) dramatically increases hepcidin expression and renders it unresponsive to inflammatory stimuli [19]. Additionally, dietary iron absorption in mice accounts for as much as 50% of the daily iron fluxes in mice fed standard chow, but only ∼5–10% in humans [20]. This increased dietary iron absorption in mice may diminish the relative contribution of iron recycling by macrophages [21] and leads to progressive iron loading. Thus, the reduction of dietary iron content in the mouse chow was designed to model iron fluxes of human homeostasis. For the slow-growing model of ovarian cancer, female mice were maintained on an iron-sufficient diet of either 20 ppm iron or 50 ppm iron.

To generate a metastatic lung cancer model, animals were injected intraperitoneally (IP) with 0.1×10^6^–0.5×10^6^ murine TC-1 or Lewis lung carcinoma (LLC) cells (ATCC, Manassas, VA). Because these are metastatic tumor cells, and we were investigating the systemic response to these cancers, we opted to perform intraperitoneal injections rather than incubating the tumor cells in their primary organ. The LLC cells were cultured as adherent cells in Dulbecco's modified Eagle media (DMEM) (Life Technologies, Grand Island, NY) supplemented with 10% fetal bovine serum (FBS) and 1% penicillin G-streptomycin, and the TC-1 cells were cultured as adherent cells in RPMI 1640 (Life Technologies, Grand Island, NY) supplemented with 10% FBS and 1% penicillin G-streptomycin. On the day of treatment, tumor cells were resuspended in phosphate buffered saline (PBS), counted with a hematocytometer, and injected in a volume of 500 µL PBS. Control mice were injected IP with 500 µL PBS. Mice were euthanized at 13–15 days, and blood, liver, and spleen were collected at necropsy.

For the mouse model of metastatic melanoma, animals were injected IP with 0.1×10^6^–0.3×10^6^ B16-F10 cells (ATCC, Manassas, VA). The B16-F10 cells were cultured as adherent cells in DMEM (Life Technologies, Grand Island, NY) supplemented with 10% FBS and 1% penicillin G-streptomycin. On the day of treatment, tumor cells were resuspended in PBS, counted with a hematocytometer, and injected in a volume of 500 µL PBS. Control mice were injected IP with 500 µL PBS. Mice were euthanized at 14 days, and blood and liver were collected at necropsy.

For peritoneally disseminated ovarian cancer, animals were injected IP with 1×10^6^ ID8 cells that were generously provided by Dr. Oliver Dorigo. The metastatic ID8 cell line was derived from spontaneous malignant transformation of C57BL/6 mouse ovarian surface epithelium cells *in vitro* [22]. The cells were cultured in DMEM supplemented with 10% FBS and 1% penicillin G-streptomycin. On the day of treatment, tumor cells were resuspended in PBS, counted with a hematocytometer, and injected in a volume of 500 µL PBS. Control mice were injected IP with 500 µL PBS. Mice were euthanized at 18.5 weeks.

To study the role of hepcidin in anemia of cancer, we used male and female hepcidin-1 knockout (HKO) mice for the TC-1 model, and female HKO mice for the ID8 model. HKO mice were originally provided to our laboratory by Dr. Sophie Vaulont [23] and were backcrossed onto the C57BL/6 background as previously described [24]. For this portion of the study, HKO mice underwent dietary conditioning to prevent the development of iron overload and maintain iron levels comparable to those of WT mice. At weaning, the HKO mice were started on reduced-iron diets (4 ppm or 20 ppm) for about 2 weeks to prevent the massive iron loading characteristic of this knockout mouse. From the time of injection, the mice were maintained on a 20 ppm iron diet for the remainder of the study. For the TC-1 model, HKO mice were injected and euthanized as described above for the WT mice. For the ID8 model, HKO mice were euthanized at 16.5 weeks.

### Measurement of iron parameters and erythropoietin

Serum iron and liver non-heme iron concentrations were measured by a colorimetric assay for iron (Sekisui Diagnostics; Lexington, MA) as previously described [20].

### Hematologic studies

Complete blood counts were obtained with a HemaVet blood analyzer (Drew Scientific; Waterbury, CT). To assess iron-restricted erythropoiesis, zinc protoporphyrin (ZPP) was measured using a hematofluorometer (AVIV; Lakewood, NJ). Reticulocytes were counted by flow cytometry. Blood (5 µl) was added to 1 ml of thiazole orange in PBS with 0.1% sodium azide (PBS-azide, BD Bioscience; San Jose, CA) and incubated at room temperature for 1–3 h. As an unstained control, blood was added to PBS-azide without thiazole orange. The percentage of red-fluorescent reticulocytes (Retic %), was measured by flow cytometry at the UCLA Jonsson Comprehensive Cancer Center (JCCC) and Center for AIDS Research Flow Cytometry Core Facility that is supported by National Institutes of Health awards CA-16042 and AI-28697, and by the JCCC, the UCLA AIDS Institute, and the David Geffen School of Medicine at UCLA. Unstained controls were used to establish a gate to exclude background fluorescence. The results are expressed as the reticulocyte product index: RPI = Retic %×Hgb/baseline Hgb.

### RNA isolation and real-time quantitative PCR

Total RNA was isolated from liver and analyzed by real-time RT-PCR as described previously [25].

Murine β-actin was used as a housekeeping mRNA control. Primers are listed in Table S1.

### Histocytopathology

Peripheral blood smears were performed using 10 µL whole blood at the time of necropsy, and prepared with Wright-Giemsa stain (Fisher; Hampton, NH).

### Statistics

SigmaStat was used for all statistical analyses (Systat Software; Point Richmond, CA). For the melanoma, lung cancer, and hepcidin KO models, normally distributed data were compared using Student *t*-test. Measurements that were not normally distributed were compared by the nonparametric Mann-Whitney rank sum test. P<0.05 was considered statistically significant. ID8 wild-type mouse data was initially analyzed using Two Way ANOVA, with ID8/saline injection and diet iron as the two variables affecting outcome. This statistical analysis showed no significant interaction effect between the two variables. The ID8 wild-type data is presented using Student *t*-test for parametric data, and Mann-Whitney rank sum test for nonparametric data. Correlation measurements were obtained by Pearson Correlation.

Both male and female WT and HKO mice were used for the three rapidly-growing cancer mouse models. Analysis of key hematologic parameters, including hemoglobin and serum iron, showed that there was no significant difference between the male and female mice. Thus, we are presenting combined male and female data.

## Results

### Mice with metastatic lung cancers develop anemia with reticulocytosis and iron restriction

**Figure 1:**
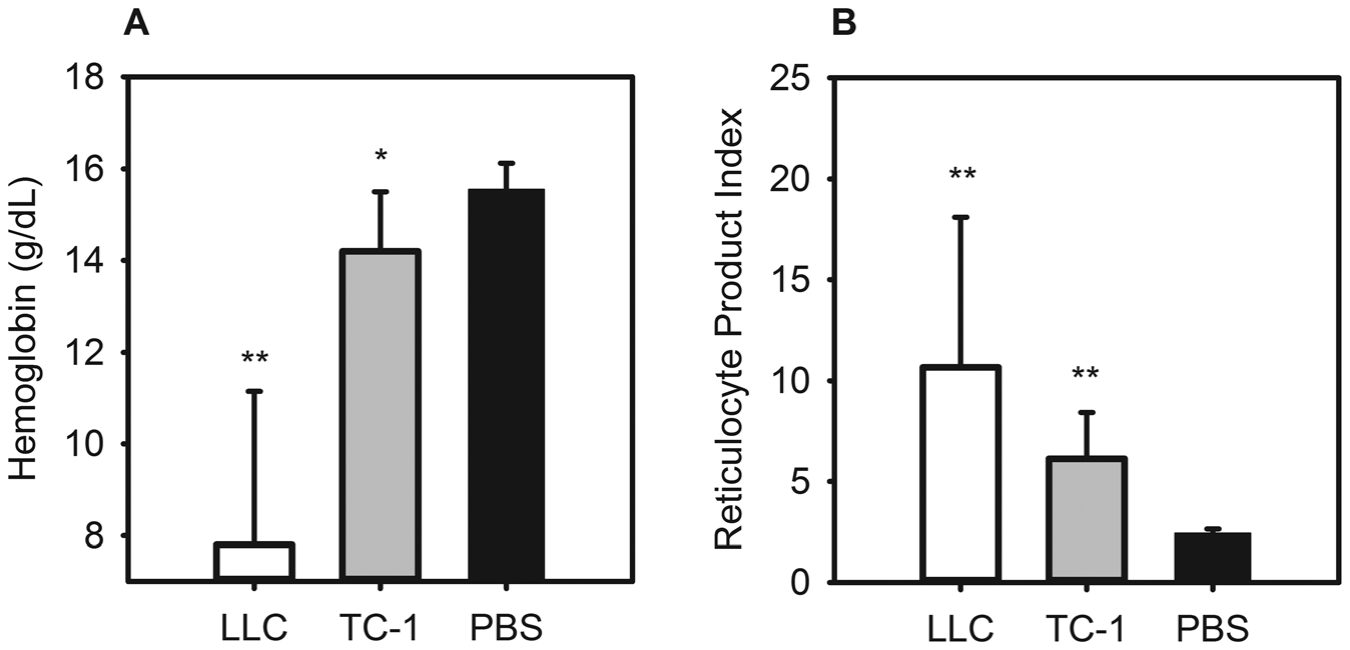
Mice with metastatic lung cancer develop anemia with reticulocytosis. C57BL/6 mice were injected intraperitoneally with 0.1×10^6^–0.5×10^6^ murine TC-1 or Lewis lung carcinoma (LLC) cells and euthanized after 14 days. Compared to controls, the tumor-bearing mice have: (A) decreased hemoglobin; (B) increased reticulocytosis. N = 8–19 mice per treatment and control group. *P<0.05, **P<0.001; P by Mann-Whitney rank sum test. Bars and error bars are median ±75^th^/25^th^ percentile.

We generated two models of metastatic lung cancer by injecting the syngeneic cancer cell lines, TC-1 and LLC, into the peritonea of mice. Control mice received PBS. Before euthanasia at 14 d after injection, all the tumor mice displayed signs consistent with systemic illness secondary to tumor load, including lethargy, poor grooming, and ascites. Necropsy revealed intraperitoneal masses in most of the mice. Hemoglobin was lower in the tumor mice as compared to controls (Fig. 1A) (LLC 7.8 g/dL vs. PBS 15.6 g/dL, P<0.001; TC-1 14.2 g/dL vs. PBS 15.6 g/dL, P = 0.029). The anemic tumor mice had increased reticulocytosis as compared to the controls (Fig. 1B) (RPI in LLC 10.7 vs. PBS 2.4, P<0.001; TC-1 6.1 vs. PBS 2.4, P<0.001). Of note, the LLC mice had lower hemoglobin levels than TC-1 mice (P<0.001) and greater reticulocytosis (P = 0.006).

**Figure 2:**
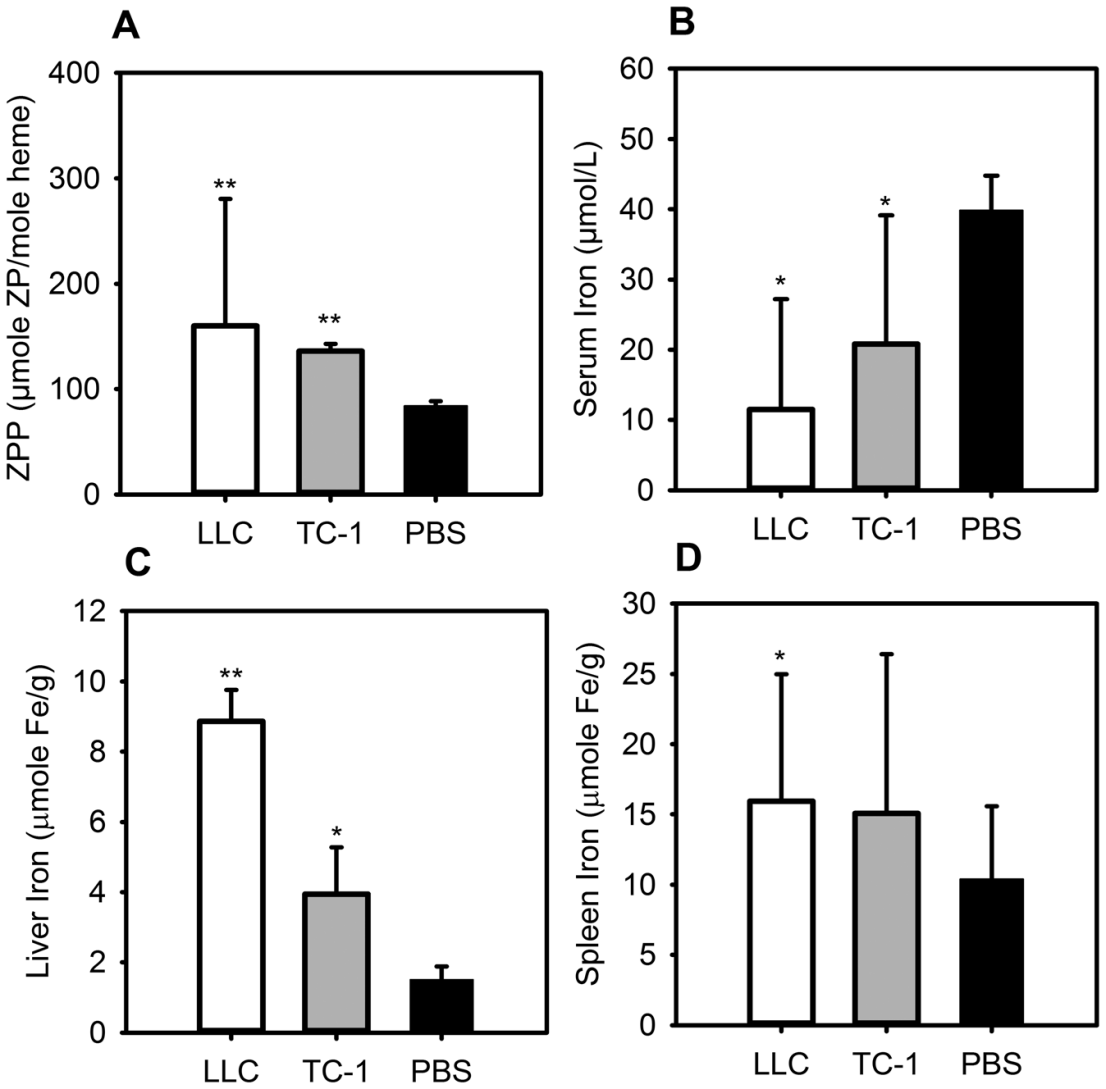
Mice with metastatic lung cancer have iron-restricted erythropoiesis with hypoferremia and increased tissue iron stores. C57BL/6 mice were injected intraperitoneally with TC-1 or LLC cells and euthanized after 14 days. Both tumor-bearing mouse models show: (A) elevated ZPP levels compared to controls, indicating iron-restricted heme synthesis; (B) hypoferremia; (C) increased liver iron. (D) The LLC-bearing mice have increased spleen iron, and the TC-1-bearing mice have a trend towards increased spleen iron, TC-1 vs. PBS, P = 0.381. N = 8–19 mice per treatment and control group. *P<0.05, **P<0.001; P by Mann-Whitney rank sum test. Bars and error bars are median ±75^th^/25^th^ percentile.

When insufficient iron is available for erythropoiesis, increased levels of zinc are incorporated into the protoporphyrin ring. Therefore, zinc protoporphyrin (ZPP) levels are a good indicator of iron-restricted erythropoiesis [26]. ZPP levels in tumor-bearing mice were increased compared to controls, indicating iron-restricted erythropoiesis (Fig. 2A) (LLC 160 vs. PBS 84, P<0.001; TC-1 136 vs. PBS 84, P<0.001). LLC mice had a trend towards higher ZPP levels than the less anemic TC-1 mice (P = 0.129). Tumor-bearing mice had hypoferremia as indicated by a serum iron assay (Fig. 2B) (LLC 11.5 µmol/L vs. PBS 39.8 µmol/L, P = 0.004; TC-1 20.8 µmol/L vs. PBS 39.8 µmol/L, P = 0.031). In order to determine whether this was due to iron sequestration, as would be seen in anemia of inflammation, rather than iron-deficiency anemia secondary to malnutrition or bleeding, total body iron stores were estimated using liver and spleen tissue iron measurements. Both lung tumor models had higher liver nonheme iron content than controls (Fig. 2C) (LLC 8.9 µmole Fe/g vs. PBS 1.5 µmole Fe/g, P<0.001; TC-1 3.9 µmole Fe/g vs. PBS 1.5 µmole Fe/g, P = 0.002). LLC mice also had higher spleen nonheme iron content than controls, and TC-1 mice had comparable spleen iron content (Fig. 2D) (LLC 15.9 µmole Fe/g vs. PBS 10.4 µmole Fe/g, P = 0.039).

### Mice with metastatic lung cancers manifest inflammation, but hepcidin is increased only in the less severe model

**Figure 3:**
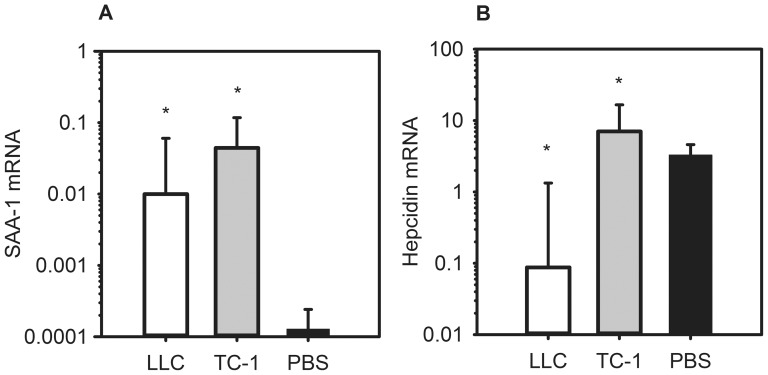
TC-1 and LLC tumor-bearing mice show evidence of systemic inflammation but only TC-1 has increased hepcidin mRNA. By day 14, (A) both tumor models have increased levels of liver SAA-1 mRNA; (B) TC-1-bearing mice have increased liver hepcidin mRNA levels compared to controls, and LLC-bearing mice have decreased hepcidin. N = 8–19 mice per treatment and control group. *P<0.05; P by Mann-Whitney rank sum test. Bars and error bars are median ±75^th^/25^th^ percentile.

In order to evaluate the contribution of inflammation and hepcidin activity to the development of anemia in our cancer models, we measured the hepatic mRNA concentrations of SAA-1 and hepcidin. SAA-1 is a hepatic acute phase reactant that is known to be increased in response to inflammation and certain cytokines, particularly IL-6 [27]. As IL-6 is thought to be an important cause of the anemia of inflammation by inducing hepcidin expression [19], we used SAA-1 measurements as an indicator of IL-6 activity. On day 14, both TC-1 and LLC mice had elevated liver SAA-1 mRNA levels as compared to controls (Fig. 3A). LLC mice had a >75-fold increase over the PBS mice (P = 0.001), and TC-1 mice had a >340-fold increase (P = 0.002). On day 14, the mildly anemic TC-1 mice had mildly elevated liver hepcidin mRNA levels as compared to controls (>2×, P = 0.012) (Figure 3B), while the more severely anemic LLC mice had lower hepcidin levels (P = 0.022), perhaps because of the counterregulatory effect on hepcidin of moderate anemia and the resulting erythropoietic stimulus [28]. To assess the influence of inflammation on hepcidin mRNA expression, we evaluated the correlation between the levels of SAA-1 mRNA and corresponding hepcidin mRNA levels in individual mice but the correlation was weak [data not shown]. Despite much higher SAA-1 levels in the tumor-bearing mice than in controls, indicative of tumor-induced inflammation, hepcidin mRNA concentrations were not increased proportionally in TC-1-bearing mice and were even decreased in LLC-bearing mice. These data suggest that other factors may counteract the effect of inflammation on hepcidin synthesis, and these could include anemia and increased erythropoiesis.

Hepcidin mRNA levels were also measured in the tumors to determine whether tumor hepcidin expression may be contributing to the development of anemia of cancer. Neither TC-1 nor LLC tumors had detectable hepcidin expression, illustrating that these tumors do not directly contribute to systemic hepcidin levels.

### Mice with melanoma develop anemia with iron-restricted erythropoiesis and inflammation

**Figure 4:**
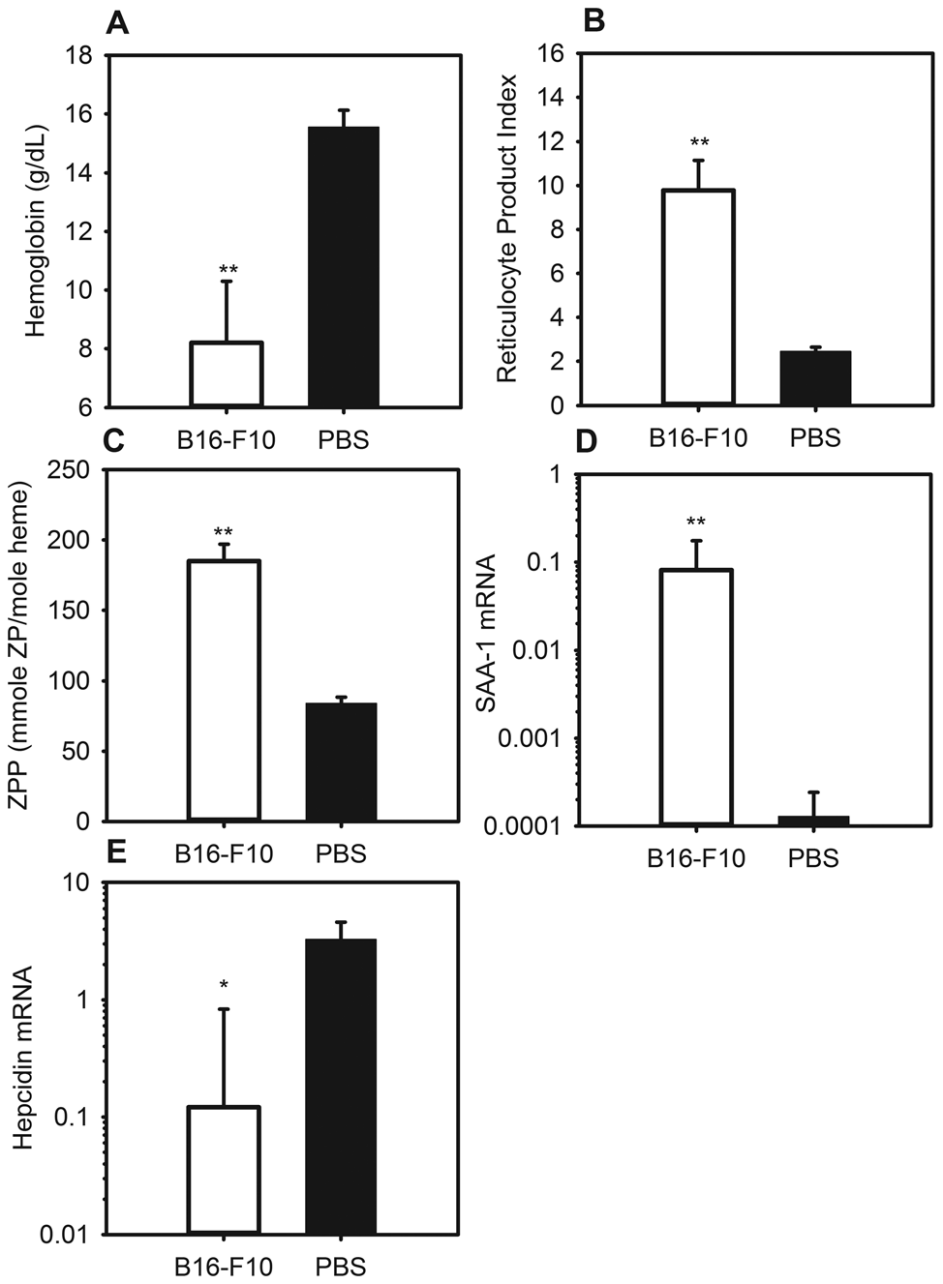
The melanoma-bearing mice develop anemia with iron-restricted erythropoiesis and inflammation. C57BL/6 mice were injected intraperitoneally with 0.1×10^6^–0.3×10^6^ B16-F10 cells, and euthanized after 14 days. The melanoma-bearing mice develop: (A) a significant anemia; (B) increased reticulocytosis compared to controls; (C) elevated zinc protoporphyrin (ZPP) levels compared to controls, indicating iron-restricted heme synthesis; (D) increased levels of liver SAA-1 mRNA, a marker of inflammation and IL-6 activity; (E) lower levels of hepcidin mRNA. N = 8–11 mice per treatment and control group. *P<0.05, **P<0.001; P by Mann-Whitney rank sum test. Bars and error bars are median ±75^th^/25^th^ percentile.

We next generated a mouse model of metastatic melanoma by injecting a melanoma cell line, B16-F10, into the peritonea of mice. Control mice received PBS. Before euthanasia 14 d after injection, the tumor mice displayed signs consistent with systemic illness and tumor burden, including lethargy and ascites. Complete blood count analysis revealed a marked anemia in the melanoma mice as compared to controls (Fig. 4A) (B16-F10 Hgb 8.2 g/dL vs. PBS Hgb 15.6 g/dL, P<0.001). The tumor mice also developed a robust reticulocytosis >4× that of their control counterparts (Fig. 4B). Melanoma mice demonstrated significant iron-restricted erythropoiesis with a >2-fold increase in ZPP levels as compared to controls (Fig. 4C).

On day 14, the melanoma mice had a dramatic increase (>600-fold) in SAA-1 mRNA as compared to controls, indicating robust inflammation (Fig. 4D). Hepcidin mRNA levels were suppressed in the tumor mice as compared to controls (Fig. 4E). Because this tumor elicited an anemia with very similar characteristics to that caused by metastatic LLC lung cancer, its effect on iron homeostasis was not characterized further.

### Mice with ovarian cancer develop iron-deficiency anemia

**Figure 5:**
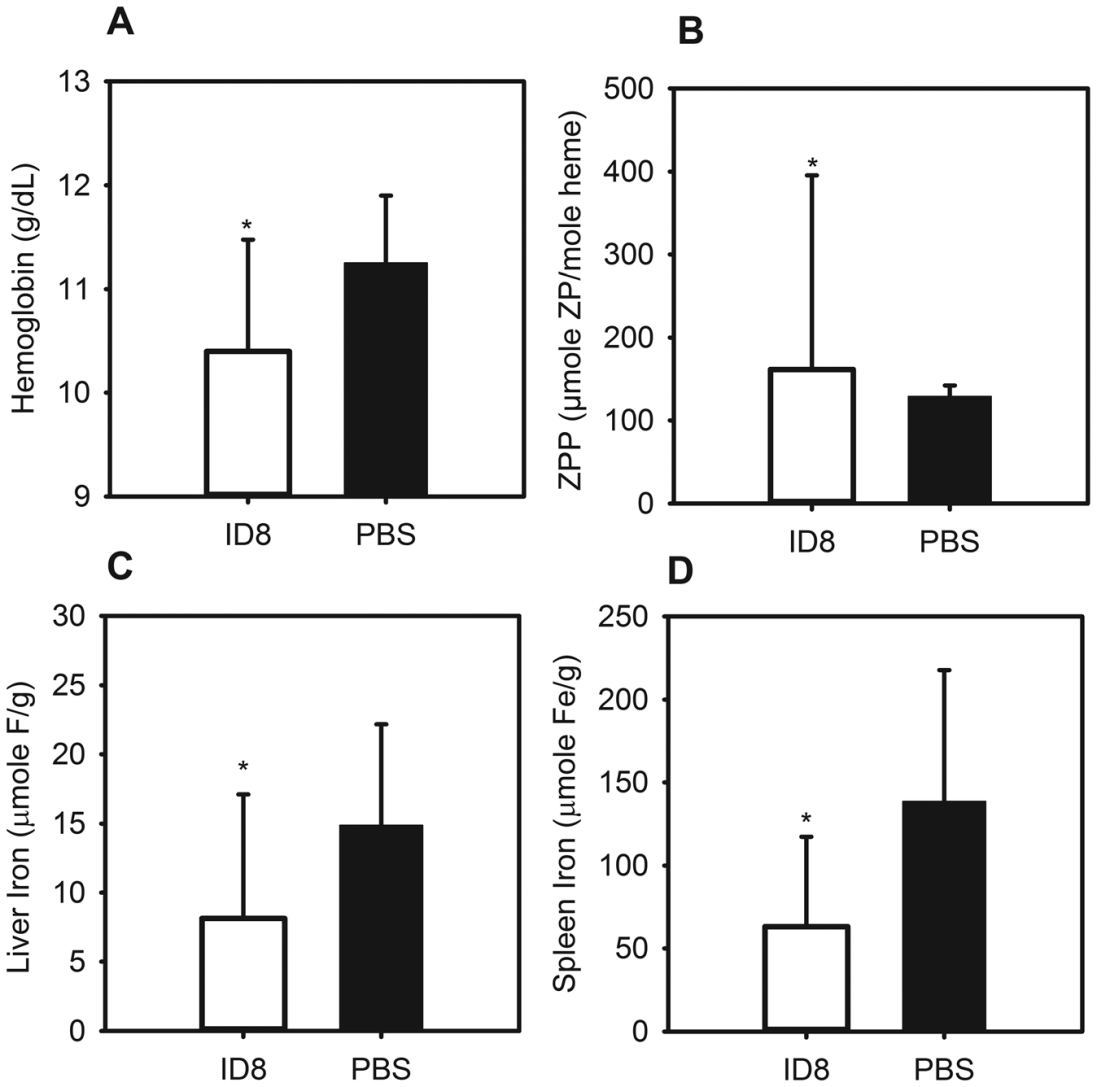
ID8-bearing mice develop iron deficiency anemia. C57BL/6 mice were injected intraperitoneally with 1×10^6^ ID8 cells and euthanized after 18.5 weeks. Compared to controls, ID8-bearing mice: (A) are more anemic; (B) have elevated ZPP levels; (C,D) have lower liver and spleen iron levels. N = 10–16 mice per treatment and control group. *P<0.05, **P<0.001; P by Mann-Whitney rank-sum test (A,B) or student *t*-test (C,D). Bars and error bars are median ± median ±75^th^/25^th^ percentile (A,B) or mean ± SD (C,D).

We also analyzed the development of anemia in mice with a slow-growing ovarian cancer by injecting the syngeneic ovarian cancer cell line ID8 into the peritonea of mice. The ID8-injected and PBS-injected control mice were sacrificed 18.5 weeks after the date of injection. At this time, all the tumor-bearing mice displayed signs consistent with systemic illness, including lethargy, poor grooming, and ascites. Necropsy revealed visible intraperitoneal masses in the majority of the mice with bloody ascites in many. Hemoglobin was lower in tumor-bearing mice as compared to controls (Fig. 5A) (ID8 10.4 g/dL vs. PBS 11.25 g/dL, P = 0.040). PBS mice themselves had moderately decreased hemoglobin levels, likely as a consequence of having been fed a reduced-iron diet for 18 weeks. The tumor-bearing mice had elevated ZPP levels as compared to controls, indicating iron-restricted erythropoiesis (Fig. 5B) (ID8 162 vs. PBS 129, P = 0.008). Total body iron stores were evaluated using liver and spleen tissue iron measurements. The ID8-bearing mice had lower liver and spleen iron content than controls (Fig. 5C,D) (ID8 liver iron 8.1 µmole Fe/g vs. PBS liver iron 14.9 µmole Fe/g, P = 0.041; ID8 spleen iron 63.1 µmole Fe/g vs. PBS spleen iron 138.5 µmole Fe/g, P = 0.011). Thus this ovarian tumor model develops anemia with frank iron deficiency, likely partially secondary to bleeding into the peritoneal cavity and impaired nutrition.

### Mice with ovarian cancer have suppressed hepcidin levels responsive to liver iron stores

**Figure 6:**
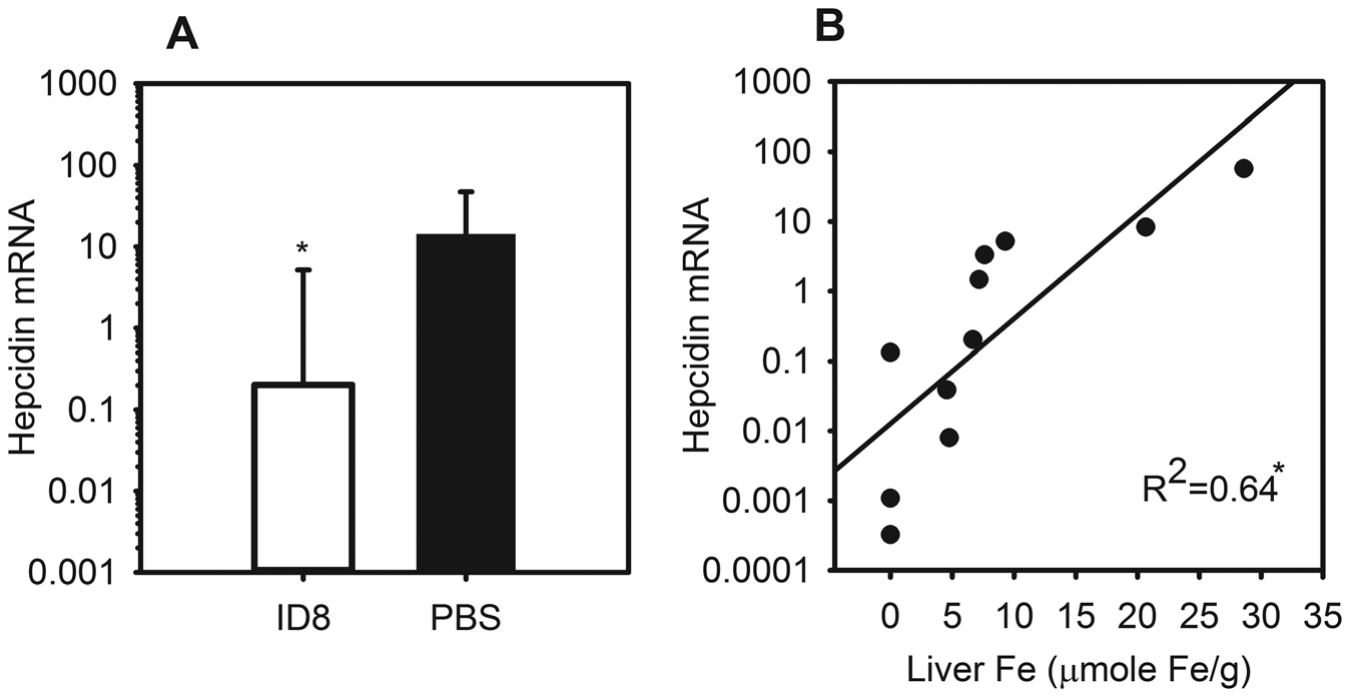
ID8-bearing mice have decreased liver hepcidin mRNA responsive to decreased iron stores. At 18.5 weeks, ID8-bearing mice compared to controls have (A) suppressed hepcidin mRNA; (B) significant positive correlation between liver iron stores and hepcidin mRNA expression. N = 11–16 mice per treatment and control group. *P<0.05; P Mann-Whitney rank sum test in (A), and by Pearson Correlation in (B). Bars and error bars are median ±75^th^/25^th^ percentile.

At 18.5 weeks, ID8 mice had depressed liver hepcidin mRNA levels as compared to controls (Fig. 6A) (ID8 0.20 vs. PBS 14.08, P = 0.013), as well as comparable SAA-1 levels to controls (Fig. S1) (P = 0.361), indicating that inflammation and hepcidin do not play a major role in the development of this anemia. In addition, figure 6B shows a significant positive correlation between hepcidin and liver iron levels, which indicates appropriate hepcidin suppression with the development of iron-deficiency anemia in this ovarian tumor model (ID8 hepcidin vs. liver iron, R^2^ = 0.64, P = 0.017).

### The development of anemia in the mouse tumor models is not secondary to microangiopathic hemolysis

**Figure 7:**
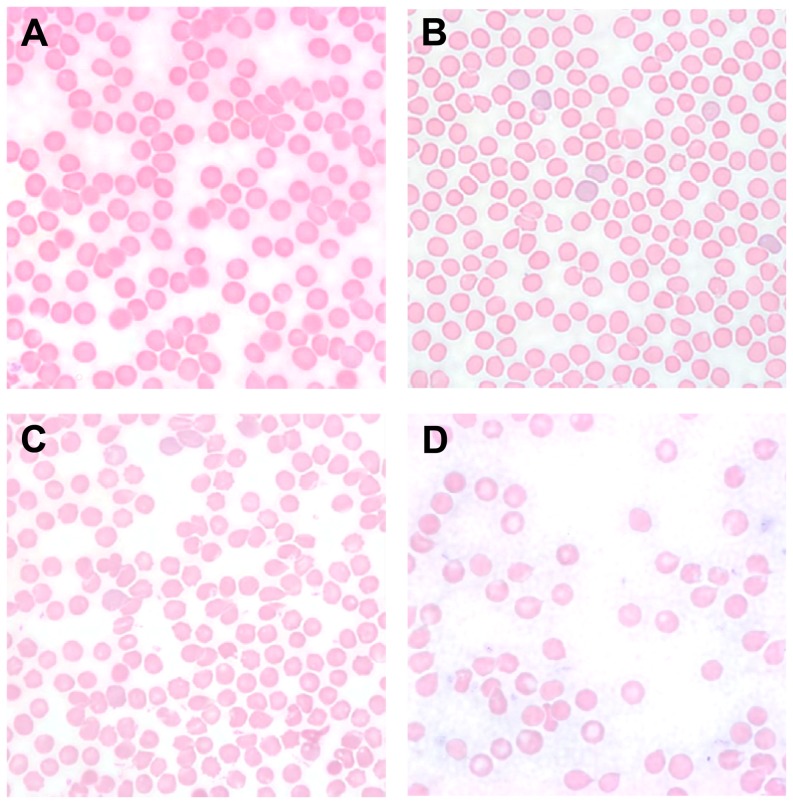
Peripheral blood smears from wild-type tumor and control mice show no significant schistocytosis. (A) PBS control (B) TC-1 (C) LLC (D) ID8.

The list of potential causes of anemia in cancer models includes microangiopathic hemolysis or shearing of erythrocytes, which can also be seen in inflammatory anemias [6]. The peripheral blood smears of the tumor-bearing mice showed no schistocytes or erythrocyte fragments. Figure 7B shows a nearly normal blood smear of the TC-1 model, with minimal membrane irregularities. Figures 7C and 7D show that the more anemic LLC and ID8 models do have more severe erythrocyte abnormalities, including teardrop cells and central pallor, but again without a significant number of schistocytes that would indicate a contribution of microangiopathic hemolysis to anemia. [29]

### Anemia is not attenuated in hepcidin-ablated TC-1- or ID8-bearing mice

**Figure 8:**
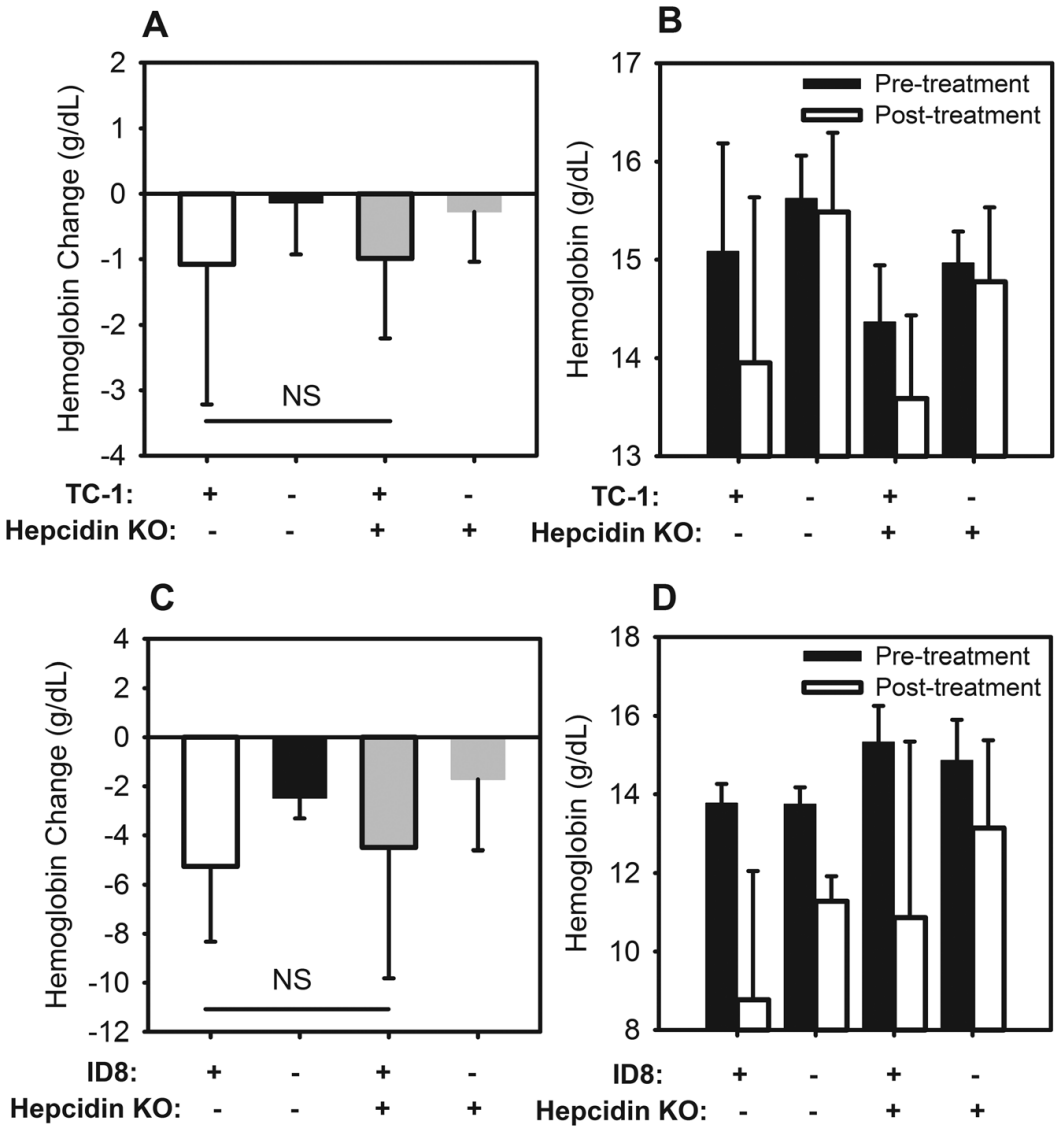
Tumor-induced hemoglobin decrease in TC-1 or ID8 -bearing mice is not affected by hepcidin ablation. (A) By day 14, there is no significant difference between the hemoglobin changes in TC-1-bearing wild-type and hepcidin knockout mice (P = 0.908). (B) Absolute hemoglobin levels of mice shown in A. (C) By 18.5 weeks, there is no significant difference between the hemoglobin changes of ID8-bearing wild-type and hepcidin knockout mice (P = 0.739). (D) Absolute hemoglobin levels of mice shown in C. N = 4–19 mice per treatment and control group. P by t-test. Bars and error bars are mean ± SD.

In order to test whether hepcidin contributes to the development of anemia in tumor-bearing mice, we injected two of the tumor cell lines into hepcidin knockout mice. At day 14 after TC-1 injection, there was no significant difference between the hemoglobin decreases of wild-type and hepcidin knockout mice (Fig. 8A) (WT TC-1 −1.08 g/dL vs. HKO TC-1 −0.99 g/dL, P = 0.908). Although TC-1 mice had mildly elevated levels of hepcidin, hepcidin is clearly not essential for the development of this anemia. Taken together with the increase in SAA-1 levels, these data point to a hepcidin-independent inflammatory mechanism for anemia.

To examine whether hepcidin expression is necessary for the development of iron deficiency in a more chronic model, we compared the development of anemia in WT and hepcidin knockout mice bearing ID8 ovarian cancer. At 18.5 weeks, there was no significant difference between the hemoglobin decreases of wild-type and hepcidin knockout mice treated with ID8 (Fig. 8C) (WT ID8 −5.26 g/dL vs. HKO ID8 −4.50 g/dL, P = 0.739). These data confirm our findings that ID8-bearing mice develop anemia in response to bleeding and nutritional iron deficiency, rather than a hepcidin-mediated inflammatory mechanism.

## Discussion

Anemia of cancer (AC) is an often overlooked but important manifestation of a malignancy. Even mild anemia has been shown to adversely affect patient-reported quality of life (QOL) parameters [30]. Although a decreased hemoglobin is only one factor in the development of depressed QOL measurements in cancer patients, the improvement of anemia has been shown to have a significant and direct correlation with overall QOL [31]. The improvement in QOL with anemia treatment persisted regardless of partial or complete response to any chemotherapy regimen. Additionally, AC has been shown to be an independent negative prognostic factor for survival time of cancer patients [32].

Despite the adverse effects of AC, it is under-recognized and under-treated by physicians [33], in part because of the lack of safe and effective therapies. Among potential treatments, red blood cell transfusions are associated with immunosuppressive effects, infection transmission, and transfusion reactions [34]. Erythropoiesis-stimulating agents, such as recombinant human erythropoietin, have been associated with higher mortality rates in patients with head and neck cancer, breast cancer, and non-small cell lung cancer [35]. Preclinical models should facilitate the development of safer and more effective treatments.

In the sparse literature on the role of hepcidin in AC, there is some evidence that inflammation and increased hepcidin concentrations could contribute to AC. Butterfield et al. demonstrated that serum hepcidin levels were markedly elevated in human patients with hematologic or nonhematologic cancers, including melanoma and lung cancers [36]. Another study of anemic cancer patients by Ukarma et al. [37] demonstrated an inverse relationship between hepcidin levels and a hemoglobin response to epoetin therapy. This relationship between epoetin resistance and increased hepcidin indicates a potential role of hepcidin in the mechanism of AC development. Sasu et al. further stratified anemic cancer patients into those with inflammatory, mixed, and iron-deficiency anemias, using hematologic parameters and C-reactive protein levels [38]. This stratification showed that the inflamed patients had the highest levels of serum hepcidin, followed by the patients with the mixed anemia. There was also a direct correlation between the inflammatory markers (ferritin and CRP) and hepcidin in the AC patients, suggesting a role of inflammation-induced hepcidin increase in the pathogenesis of AC in a subset of cancer patients with prominent inflammation.

We recently published a detailed characterization of a mouse model that helped define the contribution of hepcidin in anemia of inflammation (AI) [39]. After a single intraperitoneal injection of heat-killed *Brucella abortus*, the mice developed a severe anemia with the characteristics of AI: increased SAA-1 and hepcidin, and iron restriction with increased tissue iron stores. Importantly, hepcidin ablation resulted in a significantly milder anemia with faster recovery of hemoglobin to normal. This work demonstrated the significant contribution of hepcidin to the development of AI in this mouse model.

Here we analyzed four mouse models of AC in order to investigate the roles of inflammation and hepcidin. The findings are summarized in Table 1. All three of the rapidly-growing models developed an anemia that shares some of the characteristics of AI: iron-restricted erythropoiesis, intact tissue iron stores, and inflammation. Of the two lung cancer models, the LLC mice had significantly worse anemia, iron restriction, and increase in liver iron stores, all despite an apparently lower hepcidin level than the TC-1 mice. However, we hypothesize that an earlier time point would show an increased hepcidin level in the LLC mice, followed by hepcidin suppression secondary to active erythropoiesis, as evidenced by the robust reticulocytosis seen at 14 days. Another possible etiology for the lower hemoglobin in LLC mice would be shortened erythrocyte lifespan. While the peripheral blood smears did not show frank hemolysis, there were increased erythrocyte membrane abnormalities in LLC mice that could target them for early destruction by phagocytosis.

**Table 1:** Summary of Mouse Models.

Tumor	Time Course (weeks)	Terminal Hgb (g/dL)	Inflammation	Iron Restriction	Iron Stores	Hepcidin Level	Effect of hepcidin ablation
**TC-1**	2	14.2	Severe	Present	Increased	Elevated	None
**LLC**	2	7.8	Moderate	Present	Increased	Low	—
**B16-F10**	2	8.2	Severe	Present	—	Low	—
**ID8**	18.5	10.4	Absent	Present	Decreased	Low	None

As TC-1 mice were the only group to have elevated hepcidin levels despite the suppressive effect of accelerated erythropoiesis on hepcidin, we hypothesized that the inflammatory anemia of this model would be reversible by hepcidin ablation. However, there was no difference in the development of anemia in the hepcidin-1 KO mice. This points to predominantly hepcidin-independent factors in anemia development in these mice, which could include erythropoietic suppression and decreased erythrocyte lifespan.

The limitation of our study is that only a single time point, at which the mice had advanced cancer, was analyzed for each AC model. We cannot, therefore, eliminate the possibility that hepcidin may play a minor role in the development of AC earlier in the course of the disease. In addition, different mouse models of AC are likely to display a wide spectrum of hepcidin involvement, as is true in human cancers. While our specific models do not show a vital role of hepcidin in the development of AC, the characterization of different models could show stronger hepcidin involvement.

The slow-growing model of ovarian cancer developed a frank iron-deficiency anemia, likely secondary to hemorrhagic ascites and long-term anorexia. In addition, SAA-1 levels were not elevated, confirming that these mice developed an iron-deficiency anemia rather than AI. Unsurprisingly, repeating this model in hepcidin KO mice yielded no difference in the development of anemia confirming the lack of hepcidin involvement in this model of AC.

Much is yet to be understood about the development of anemia in cancer patients. The characterization and manipulation of mouse models of cancer should be useful for the exploration of mechanisms and potential therapeutics for AC.

## Supporting Information

Figure S1 **SAA-1 expression in ovarian tumor-bearing mice.** By 18.5 weeks, the ID8-bearing and control mice have comparable SAA-1 levels (P = 0.361). N = 11–16 mice per treatment and control group. P by Mann-Whitney rank sum test. Bars and error bars are median ±75^th^/25^th^ percentile.
(TIF)

Table S1 **PCR Primers.**
(TIF)
