# Supplementary figures and images for: Mouse Models of Anemia of Cancer
Source: PLoS One. 2014 Mar 28;9(3):e93283. doi: 10.1371/journal.pone.0093283 (PMC3969362; doi:10.1371/journal.pone.0093283)

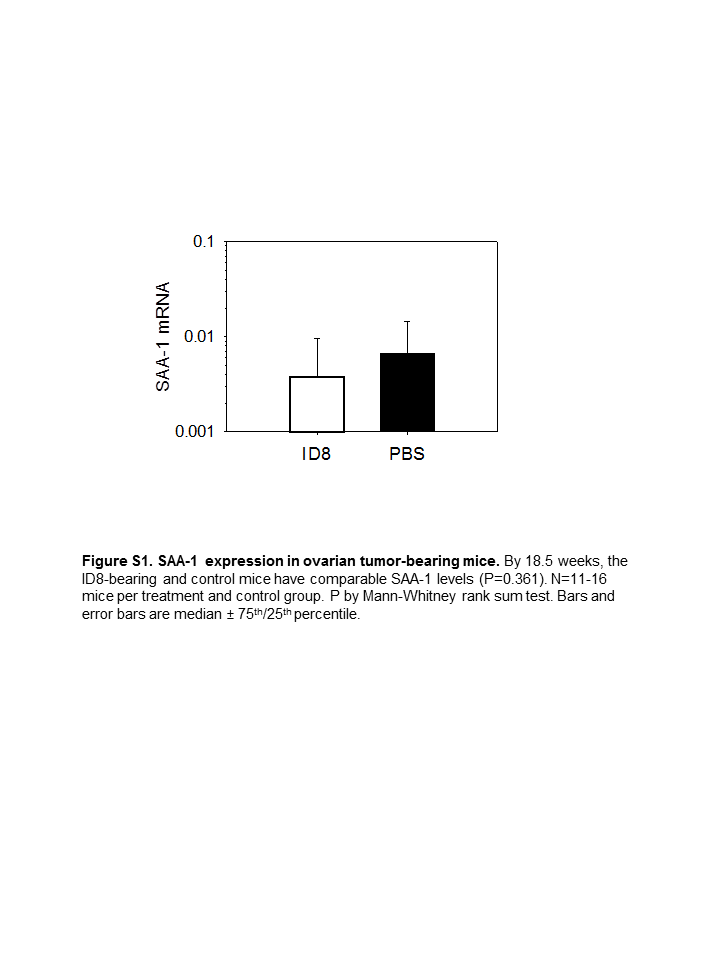

Supplement: Figure S1 — SAA-1 expression in ovarian tumor-bearing mice. By 18.5 weeks, the ID8-bearing and control mice have comparable SAA-1 levels (P = 0.361). N = 11–16 mice per treatment and control group. P by Mann-Whitney rank sum test. Bars and error bars are median ±75th/25th percentile. (TIF) [file pone.0093283.s001.tif]
